# Performance Analysis of a Proton Exchange Membrane Fuel Cell Based Syngas

**DOI:** 10.3390/e21010085

**Published:** 2019-01-18

**Authors:** Xiuqin Zhang, Qiubao Lin, Huiying Liu, Xiaowei Chen, Sunqing Su, Meng Ni

**Affiliations:** 1 Department of Physics, Jimei University, Xiamen 361021, China; 2 Department of Building and Real Estate, The Hong Kong Polytechnic University, Hong Kong, China

**Keywords:** PEM fuel cell, syngas, steam reforming, combustion, performance analysis

## Abstract

External chemical reactors for steam reforming and water gas shift reactions are needed for a proton exchange membrane (PEM) fuel cell system using syngas fuel. For the preheating of syngas and stable steam reforming reaction at 600 °C, residual hydrogen from a fuel cell and a certain amount of additional syngas are burned. The combustion temperature is calculated and the molar ratio of the syngas into burner and steam reformer is determined. Based on thermodynamics and electrochemistry, the electric power density and energy conversion efficiency of a PEM fuel cell based syngas are expressed. The effects of the temperature, the hydrogen utilization factor at the anode, and the molar ratio of the syngas into burner and steam reformer on the performance of a PEM fuel cell are discussed. To achieve the maximum power density or efficiency, the key parameters are determined. This manuscript presents the detailed operating process of a PEM fuel cell, the allocation of the syngas for combustion and electric generation, and the feasibility of a PEM fuel cell using syngas.

## 1. Introduction 

The hydrogen fuel cell is a promising technology for electric vehicle applications [1]. Compared with battery-based electric vehicles, hydrogen fuel cell-based electric vehicles can easily deliver a much higher range, and the fueling of hydrogen only takes a few minutes. However, it is still challenging to store hydrogen in vehicles with small volume and mass. The currently used hydrogen cylinder is bulky while the metal hydride is relatively heavy. One option to solve the hydrogen storage problem is to supply hydrogen onboard by the steam reforming of ethanol [2,3] or diesel [4]. This option has been demonstrated to be feasible and is practically used. However, hydrogen production from nonrenewable resources results in pollutant emission from the life cycle point of view. Biomass is an important renewable resource and is carbon neutral. The use of biomass-derived fuel in fuel cell vehicles can achieve green transportation with minimal pollutant emission. When biomass-derived fuel is used in a proton exchange membrane (PEM) fuel cell, the syngas production [5], steam reforming, and water gas shift reactions are most necessary to provide pure hydrogen. Although a recent study showed that it is possible to directly use biomass in a fuel cell without converting biomass into hydrogen rich gases [6], the power density is too low for practical applications. Two conversion paths, anaerobic digestion and gasification, are available to convert biomass into hydrogen-rich gases, which contains hydrogen, methane, carbon monoxide, carbon dioxide, and other gases [7]. As methane cannot be electrochemically oxidized in the PEM fuel cell and the carbon monoxide can easily poison the noble metal catalyst in the fuel cell, the further steam reforming and the water gas shift are necessary to convert the residual methane and carbon monoxide to hydrogen, respectively. As the steam reforming reaction is endothermic, the addition of CaO into glycerol steam reforming is proposed [8], since the conversion of CaO into CaCO_3_ can provide enough heat for the endothermic reaction. However, the new product CaCO_3_ may cover the catalyst of the steam reforming reaction and thus limits the long-term durability. The high-temperature exhaust heat from a Stirling engine can be used for steam reforming [9], but the operation of the PEM fuel cell is dependent on the engine. 

It is better if the steam reforming and the combustion of residual hydrogen from a PEM fuel cell are integrated. The combustion of residual fuel from a fuel cell and directly fed fuel of a burner can generate enough heat for the reforming [10]. A membrane reactor of reforming is employed for the improvement of the system efficiency [11]. The effect of the molar ratio of steam and hydrocarbon on the steam reforming product [12] and system efficiency [13] is present. The molar ratio of the fuel into the burner and steam reformer has almost not been discussed, and it is considered as a parameter in this paper which will affect the performance of the system.

To improve the performance of a PEM fuel cell, various strategies have been proposed. A gas diffusion layer with micropores of the PEM fuel cell is suggested [14] because the low thermal conductivity of it can accelerate the transportation of water while the operating temperature of the fuel cell may be unstable. The addition of silicates to the proton exchange membrane will improve the thermal conductivity, water uptake, and proton conductivity of the membrane [15]. The work in Reference [16] exhibits high water uptake and proton conductivity of the activated carbon/Nafion hybrid composite. The ohmic polarization loss is influenced by the proton conductivity and gas humidity [17]. If there is analytic relation between the proton conductivity and composition of the membrane, the performance of a PEM fuel cell can be optimized. The concentration polarization loss mainly occurs in the cathode [18]. The flow field of the bipolar plate is designed to reduce the loss [19]. The mathematical expressions of activation, ohm, and concentration overpotentials in engineering are used in this study.

## 2. The Auxiliary Systems outside a PEM Fuel Cell 

**Figure 1:**
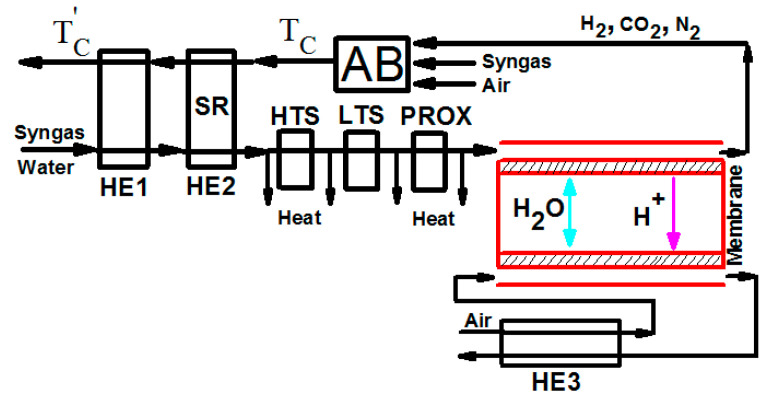
The schematic diagram of a proton exchange membrane (PEM) fuel cell based syngas, where abbreviations of HE1, HE2, and HE3 are heat exchangers, SR is a steam reformer, HTS and LTS are water gas shift reactions, PROX is a preferential oxidizer, and AB is an auxiliary burner.

The schematic diagram of the system is shown in Figure 1, where the syngas and water are preheated from 25 °C to 600 °C in heat exchanger 1 (HE1); the steam reforming reaction of methane and water vapor in the gases is taken place in heat exchanger 2 (HE2); and the carbon monoxide is eliminated after passing the high-temperature water gas shift (HTS), the low-temperature water gas shift (LTS), and the preferential oxidation reactions (PROX). The air flowing into the cathode of a fuel cell is preheated by the gases leaving the fuel cell in heat exchanger 3 (HE3). The residual hydrogen leaving the anode of a fuel cell and a certain amount of additional syngas are burned in the after burner (AB); the high-temperature combustion product is used to supply enough heat for the preheating and steam reforming reactions in HE1 and HE2, respectively. 

### 2.1. The Heat Needed in HE1 and HE2

The water gas shift and preferential oxidation reactions are exothermal; the heat about them will not be discussed. Assuming the heat released from the residual air and water leaving the cathode is enough for the preheating of air flowing into the fuel cell, the process of heat transfer in HE3 will also not be discussed.

If the mole flow rate of syngas into HE1 is n (mol·s^−1^) and the amount of water in the flow is twice of that needed for the steam reforming and water gas shift reactions, the steam reforming and water gas shift reactions are, respectively,

(1) $${\mathrm{CH}}_{4}+H_{2}O↔\mathrm{CO}+3H_{2}\;\Delta h>0$$

and

(2) $$\mathrm{CO}+H_{2}O↔{\mathrm{CO}}_{2}+H_{2}\;\Delta h<0$$

where Δh is the enthalpy change of gases if one mole $CH_{4}$ is consumed in Equation (1) or one mole CO is consumed in Equation (2). The mole rate of water added in the syngas is

(3) $$n_{H_{2}O}=2n(2x_{{\mathrm{CH}}_{4}}+x_{\mathrm{CO}})-nx_{H_{2}O},$$

where $x_{k}$ is the mole fraction of k in the syngas; the specific components of the syngas are shown in Table 1 [20]. The heat needed for the preheating of the syngas and water is

(4) $${\dot{Q}}_{1}=n\sum_{k}\left(x_{k}\int_{298.15}^{873.15}C_{k,m}dt\right)+n_{H_{2}O}\int_{298.15}^{873.15}C_{H_{2}O,m}dt+2n(2x_{{\mathrm{CH}}_{4}}+x_{\mathrm{CO}})L_{H_{2}O,m},$$

where $C_{k,m}$ is the molar heat capacity of k, the value of which is shown in Table 2 [21,22]; t is temperature; and $L_{H_{2}O,m}$ is the latent heat of one mole water. The heat needed for the steam reforming reaction is

(5) $${\dot{Q}}_{2}=nx_{{\mathrm{CH}}_{4}}(3h_{H_{2}}+h_{\mathrm{CO}}-h_{{\mathrm{CH}}_{4}}-h_{H_{2}O}),$$

where $h_{k}$ is the enthalpy of k per mole at 600 °C and 1atm and is calculated as

(6) $$h_{k}=h_{k}^{0}+\int_{298.15}^{873.15}C_{k,m}dt,$$

where $h_{k}^{0}$ is the enthalpy of k per mole at 25 °C and 1 atm, the value of which is shown in Table 2.

### 2.2. The Molar Ratio of Syngas into the Burner and Steam Reformer 

The relative low temperature of a PEM fuel cell will reduce the cost of materials and will present a short start time and a fast transient response [23]. In this paper, the working temperature of the PEM fuel cell is under 100 °C and the water can be separated from the gases which will enter into the anode of the fuel cell. If CO is reacted fully in the water gas shift reactions, the gases flowing into the fuel cell is composed of $H_{2}$, ${\mathrm{CO}}_{2}$, and $N_{2}$ by combining Equations (1) and (2). 

The total electrochemical reaction in a fuel cell is

(7) $$H_{2}+0.5O_{2}\to H_{2}O+\mathrm{Electricity}.\;\Delta h<0$$

If the hydrogen utilization factor at the anode is $u_{H_{2}}$, the mole flowing rate of residual hydrogen is determined, and the total enthalpy of residual $H_{2}$, ${\mathrm{CO}}_{2}$, and $N_{2}$ leaving the fuel cell is

(8) $${\dot{H}}_{cell}=n(x_{H_{2}}+4x_{{\mathrm{CH}}_{4}}+x_{\mathrm{CO}})(1-u_{H_{2}})h_{H_{2}}(T)+n(x_{{\mathrm{CO}}_{2}}+x_{{\mathrm{CH}}_{4}}+x_{\mathrm{CO}})h_{{\mathrm{CO}}_{2}}(T)+nx_{N_{2}}h_{N_{2}}(T),$$

where $h_{k}(T)$ is the enthalpy of k per mole at temperature T and 1 atm. 

The combustion of residual hydrogen may not be enough for the preheating and steam reforming of the syngas, so extra syngas is added in the burner. The combustion equations in the burners are, respectively,

(9) $$H_{2}+0.5O_{2}\to H_{2}O,$$

(10) $${\mathrm{CH}}_{4}+2O_{2}\to {\mathrm{CO}}_{2}+2H_{2}O,$$

and

(11) $$\mathrm{CO}+0.5O_{2}\to {\mathrm{CO}}_{2}.$$

Assuming the molar ratio of the syngas into the burner and steam reformer is x and the air in the burner is just enough for the combustion, the enthalpy of gases flowing into the burner unit time is

(12) $${\dot{H}}_{in}={\dot{H}}_{cell}+nx\sum_{k}x_{k}h_{k}^{0}+\frac{1}{2}n[(x_{H_{2}}+4x_{{\mathrm{CH}}_{4}}+x_{\mathrm{CO}})(1-u_{H_{2}})+xx_{H_{2}}+xx_{\mathrm{CO}}+4xx_{{\mathrm{CH}}_{4}}](h_{O_{2}}^{0}+\frac{78}{21}h_{N_{2}}^{0})$$

Combining Equations (8)–(12), the component of the combustion product is determined. If the combustion temperature is $T_{C}$, one can obtain the enthalpy of gases leaving the burner as

(13) $$\begin{array}{l} {\dot{H}}_{out}=n[(x_{H_{2}}+4x_{{\mathrm{CH}}_{4}}+x_{\mathrm{CO}})(1-u_{H_{2}})+xx_{H_{2}}+xx_{H_{2}O}+2xx_{{\mathrm{CH}}_{4}}]h_{H_{2}O}^{}(T_{C})+\\ n(1+x)(x_{{\mathrm{CO}}_{2}}+x_{{\mathrm{CH}}_{4}}+x_{\mathrm{CO}})h_{{\mathrm{CO}}_{2}}^{}(T_{C})+n(1+x)x_{N_{2}}h_{N_{2}}(T_{C})+\frac{1}{2}n[(x_{H_{2}}+4x_{{\mathrm{CH}}_{4}}+x_{\mathrm{CO}})(1-u_{H_{2}})+\\ xx_{H_{2}}+xx_{\mathrm{CO}}+4xx_{{\mathrm{CH}}_{4}}]\frac{78}{21}h_{N_{2}}^{}(T_{C}). \end{array}$$

Assuming the burner is adiabatic, the enthalpy of gases flowing into the burner is equal to that leaving the burner:

(14) $${\dot{H}}_{in}={\dot{H}}_{out}.$$

Combining Equations (8) and (12)–(14), x is a function of $u_{H_{2}}$, T, and $T_{C}$. $T_{C}$ is not random; it should be larger than 600 °C, and the heat released by the combustion product must be enough for the preheating and steam reforming reaction in HE1 and HE2 as

(15) $$\begin{array}{l} n[(x_{H_{2}}+4x_{{\mathrm{CH}}_{4}}+x_{\mathrm{CO}})(1-u_{H_{2}})+xx_{H_{2}}+xx_{H_{2}O}+2xx_{{\mathrm{CH}}_{4}}](\int_{T_{C}^{\prime }}^{T_{C}}C_{H_{2}O,m}dt+L_{H_{2}O,m})+\\ n(1+x)(x_{{\mathrm{CO}}_{2}}+x_{{\mathrm{CH}}_{4}}+x_{\mathrm{CO}})\int_{T_{C}^{\prime }}^{T_{C}}C_{{\mathrm{CO}}_{2},m}dt+n(1+x)x_{N_{2}}\int_{T_{C}^{\prime }}^{T_{C}}C_{N_{2},m}dt+\\ \frac{1}{2}n[(x_{H_{2}}+4x_{{\mathrm{CH}}_{4}}+x_{\mathrm{CO}})(1-u_{H_{2}})+xx_{H_{2}}+xx_{\mathrm{CO}}+4xx_{{\mathrm{CH}}_{4}}]\frac{78}{21}\int_{T_{C}^{\prime }}^{T_{C}}C_{N_{2},m}dt\geq {\dot{Q}}_{1}+{\dot{Q}}_{2}, \end{array}$$

where $T_{C}^{\prime }$ is the temperature of the combustion product leaving HE1. If the rate of heat released from the combustion product is as close to that needed for the preheating and steam reforming reaction as possible, Equation (15) is another relationship between x, $u_{H_{2}}$, T, and $T_{C}$. 

**Figure 2:**
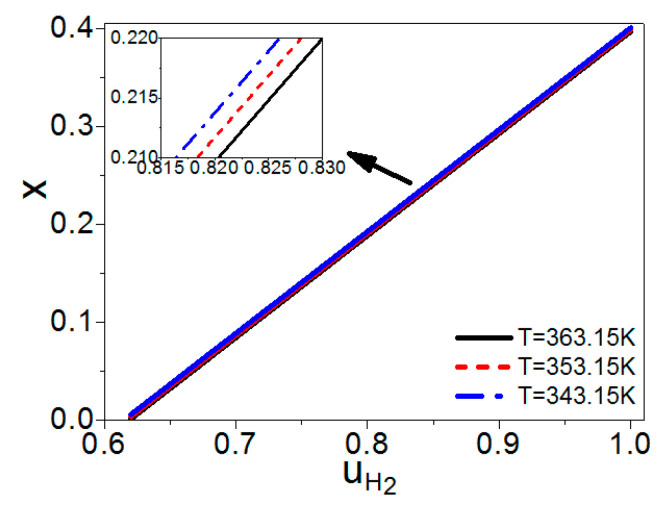
Curves of x varying with $u_{H_{2}}$, where $T_{C}^{\prime }$ = 30 °C.

**Figure 3:**
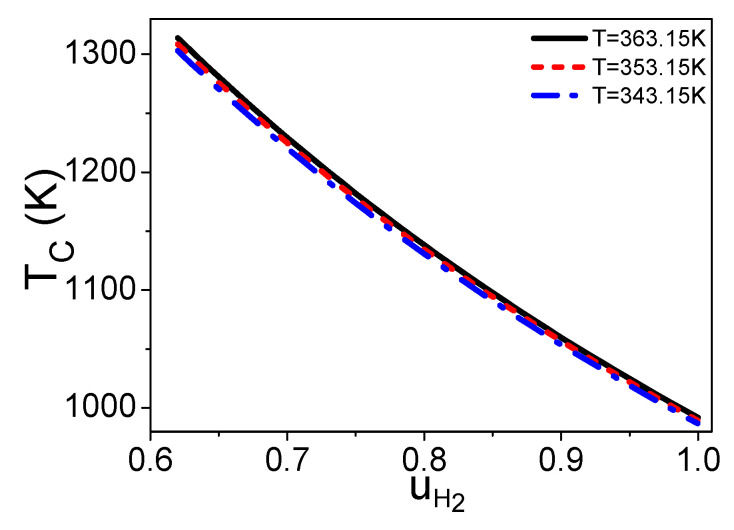
Curves of $T_{C}^{}$ varying with $u_{H_{2}}$, where $T_{C}^{\prime }$ = 30 °C.

Combining Equations (3)–(6), (8), and (12)–(15), x and $T_{C}^{}$ are both independent of n, while they are functions of $u_{H_{2}}$, T, and $T_{C}^{\prime }$. If T and $T_{C}^{\prime }$ are given, one can obtain the curves of x and $T_{C}^{}$ varying with $u_{H_{2}}$ as shown in Figure 2 and Figure 3, respectively. If the hydrogen utilization factor is smaller, there are more residual hydrogen leaving the fuel cell, the combustion temperature is higher, and the amount of syngas in the burner is less as shown in Figure 2 and Figure 3, so the cost of the steam reforming reaction is lower. However, the hydrogen utilization factor at the anode of a fuel cell will affect the electric power of the fuel cell [24]; there is a specific hydrogen utilization factor under the maximum electric power. The energy conversion efficiency is dependent on both the amount of syngas in the burner and the electric power need to be introduced and optimized. Figure 2 and Figure 3 also show that if the working temperature of the PEM fuel cell is higher, the temperature of the combustion product is higher and the amount of syngas needed for the burner is less. 

## 3. The Electric Power of a PEM Fuel Cell Based Syngas

As defined above, the molar rate of the hydrogen used in Equation (7) is

(16) $$n(x_{H_{2}}+4x_{{\mathrm{CH}}_{4}}+x_{\mathrm{CO}})u_{H_{2}}.$$

According to thermodynamics, the available energy released by the reaction is

(17) $$n(x_{H_{2}}+4x_{{\mathrm{CH}}_{4}}+x_{\mathrm{CO}})u_{H_{2}}\Delta g(T),$$

where Δg(T) is the Gibbs function change of the gases if one molar hydrogen is consumed in Equation (7), and it can be calculated as

(18) Δg(T)=Δh(T)−TΔs(T),

where Δh(T) and Δs(T) are, respectively, the enthalpy and entropy changes of the gases if one molar hydrogen is consumed in Equation (7). The enthalpy change of the gases is

(19) $$\Delta h(T)=h_{H_{2}O}(T)-h_{H_{2}}(T)-0.5h_{O_{2}}(T).$$

The entropy change of the gases is

(20) $$\Delta s(T)=s_{H_{2}O}^{0}+\int_{298.15}^{T}\frac{C_{H_{2}O,m}}{t}dt-\left(s_{H_{2}}^{0}+\int_{298.15}^{T}\frac{C_{H_{2},m}}{t}dt\right)-0.5(s_{O_{2}}^{0}+\int_{298.15}^{T}\frac{C_{O_{2},m}}{t}dt)+R\ln\frac{p_{H_{2}}p_{O_{2}}^{0.5}}{p_{H_{2}O}},$$

where $s_{k}^{0}$ is the entropy of per mole k at 25 °C and 1 atm; R is the universal gas constant; $p_{k}$ is the partial pressure of k at the electrode; the partial pressure of liquid water is 1;

(21) $$p_{H_{2}}=p_{a}(1-\frac{p_{s}}{p_{a}})/[1+\frac{x_{a}}{2}(1+\frac{\delta_{a}}{\delta_{a}-1})];$$

and

(22) $$p_{O_{2}}=p_{c}(1-\frac{p_{s}}{p_{c}})/[1+\frac{x_{c}}{2}(1+\frac{\delta_{c}}{\delta_{c}-1})],$$

where $p_{a}$ (atm) and $p_{c}$ (atm) are pressures at anode and cathode, respectively [25], $p_{s}$ (atm) is the saturation pressure of water and is dependent on the temperature [26], $x_{a}$ and $x_{c}$ are dry gas molar ratios at anode and cathode, respectively, and $\delta_{a}$ and $\delta_{c}$ are stoichiometry coefficients. 

As shown by the equivalent circuit of a PEM fuel cell in Ref. [27], there is internal resistance of a fuel cell. The heat produced by the internal resistance is

(23) $$I(V_{act}+V_{ohm}+V_{con}),$$

where I is the intensity of the electric current of the PEM fuel cell and $V_{act}$ is the activation overpotential. The general expression of it is the Butler–Vollmer equation [28]. There is another pattern reported by Tafel [29] based on the results of the experiments and is defined as

(24) $$V_{act}=\frac{RT}{n_{e}F}\frac{\left(\alpha_{a}+\alpha_{c}\right)}{\alpha_{a}\alpha_{c}}\ln\frac{i}{i_{0}},$$

where $n_{e}$ is the number of electrons transferred through the external circuit if one molecule of hydrogen is reacted; F is Faraday’s constant; $\alpha_{a}$ and $\alpha_{c}$ represent the anode and cathode charge transfer coefficients; $i=I/A_{c}$ is the current density of the PEM fuel cell; $A_{c}$ is the effective surface area of the bipolar plate; $i_{0}$ is the exchange current density of the electrodes [30] and is defined as

(25) $$i_{0}=1.27\times 10^{-8}\exp(2.06p_{O_{2}});$$

and $V_{ohm}$ and $V_{con}$ are ohm and concentration overpotentials [31]. The expressions of them are [27]

(26) $$V_{ohm}=i\frac{\delta_{mem}}{\sigma_{mem}}$$

and

(27) $$V_{con}=i{\left(\beta_{1}\frac{i}{i_{L}}\right)}^{\beta_{2}},$$

respectively, where $\delta_{mem}$ is the thickness of membrane and $\sigma_{mem}$ is the membrane conductivity [27]. It can be defined as

(28) $$\sigma_{mem}=(0.005139\mu_{mem}-0.003260)\exp[1268(\frac{1}{303}-\frac{1}{T})],$$

where $\mu_{mem}$ is the water content and is determined by water vapor activity, $i_{L}$ is the limiting current density, $\beta_{1}$ is dependent on the partial pressure of oxygen and the temperature at the cathode [30], and $\beta_{2}$ is constant.

According to energy conservation, the electric power of a PEM fuel cell is

(29) $$P_{e}=-n(x_{H_{2}}+4x_{{\mathrm{CH}}_{4}}+x_{\mathrm{CO}})u_{H_{2}}\Delta g(T)-I(V_{act}+V_{ohm}+V_{con}).$$

Based on Faraday’s law, the intensity of the electric current in Equation (29) can be presented as

(30) $$I=n(x_{H_{2}}+4x_{{\mathrm{CH}}_{4}}+x_{\mathrm{CO}})u_{H_{2}}n_{e}F.$$

**Figure 4:**
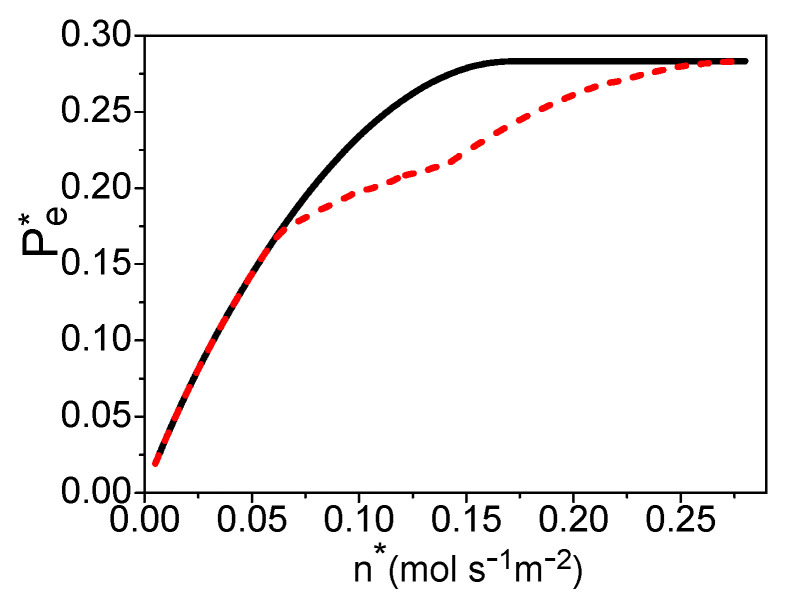
The power density varying with $n^{*}$, where T = 70 °C and $T_{C}^{\prime }$ = 30 °C. The solid and dash curves are, respectively, the maximum power density and the power density under the maximum efficiency.

**Figure 5:**
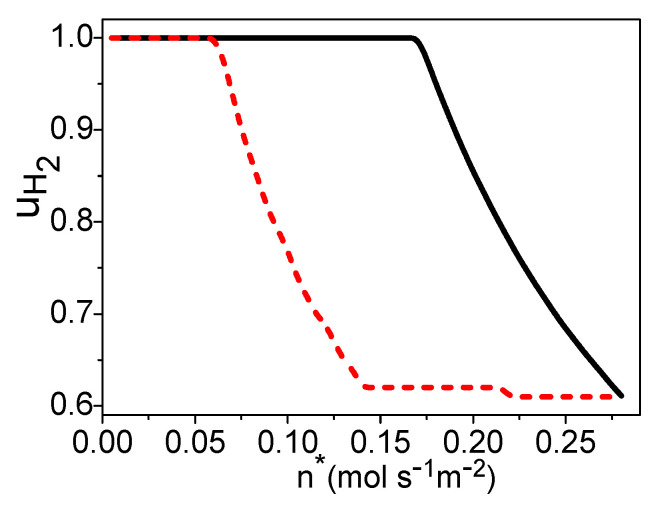
The hydrogen utilization factor varying with $n^{*}$, where T = 70 °C and $T_{C}^{\prime }$ = 30 °C. The solid and dash curves are, respectively, the hydrogen utilization factor under the maximum power density and the maximum efficiency.

Combining Equations (18)–(30) and Table 3 [25,30,32], the electric power density “$P_{e}^{*}=P_{e}/A_{c}$” is dependent on “$n^{*}=n/A_{c}$”, $u_{H_{2}}$, and T. If T is given, one can obtain the maximum power density varying with $n^{*}$ by optimizing the hydrogen utilization factor, as shown by the solid curve in Figure 4. The hydrogen utilization factor under the maximum power density is shown by the solid curve in Figure 5. There is a certain electric current density under the maximum power density of the PEM fuel cell [24]. This means that there is an optimal product of $n^{*}$ and $u_{H_{2}}$ from Equation (30). When $n^{*}$ is smaller than 0.17 (mol·s^−1^·m^−2^), the product of $n^{*}$ and $u_{H_{2}}$ is always smaller than the optimum value, even though $u_{H_{2}}$= 1 in Figure 5 and the electric power density of the PEM fuel cell is a monotonous increasing function of the molar flow rate of the syngas in Figure 4. When $n^{*}\geq$ 0.17 (mol·s^−1^·m^−2^), the hydrogen utilization factor is decreasing with the increase of the molar flow rate of syngas to reach the optimal product of them in Figure 5, and the electric power density is constant in Figure 4. 

**Figure 6:**
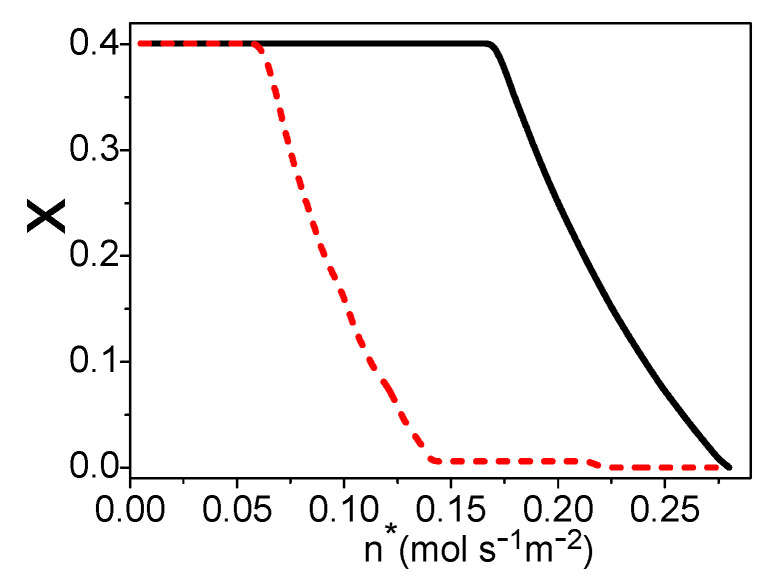
Curves of x varying with $n^{*}$, where T = 70 °C and $T_{C}^{\prime }$ = 30 °C. The solid and dash curves are x under the maximum power density and the maximum efficiency, respectively.

There is a linear relationship between x and $u_{H_{2}}$ in Figure 2, so one can also obtain *x* under the maximum power density, as shown by the solid curve in Figure 6. The graphs of Figure 5 and Figure 6 are similar. When there is no residual hydrogen from the fuel cell, the molar ratio of the syngas into the burner and steam reformer is 0.4. As the amount of residual hydrogen increases, the molar ratio x is decreasing. When the hydrogen utilization factor is about 0.61, there is no need to add syngas into the burner. As discussed in Section 2.2, the total energy conversion efficiency of the hybrid system of a PEM fuel cell and steam reformer is dependent on the hydrogen utilization factor at the anode of the fuel cell, so the efficiency of the hybrid system will be further optimized.

## 4. The Total Energy Conversion Efficiency

**Figure 7:**
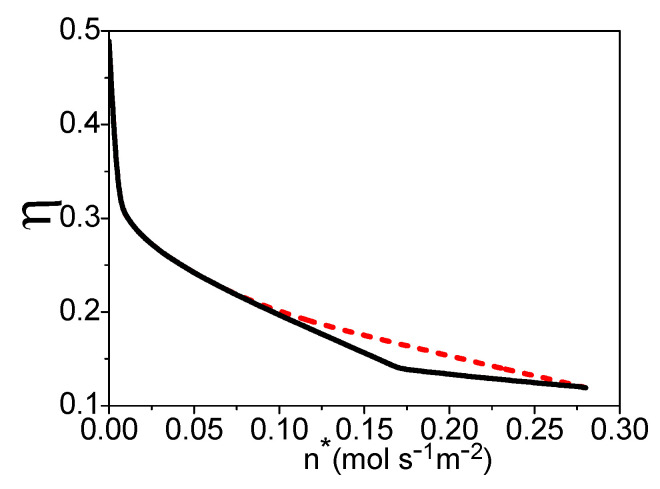
The efficiency varying with $n^{*}$, where T = 70 °C and $T_{C}^{\prime }$ = 30 °C. The dash and solid curves are, respectively, the maximum efficiency and the efficiency under the maximum power density.

The energy conversion efficiency of the hybrid system of a PEM fuel cell and steam reformer is

(31) $$\begin{array}{ll} \eta & =\frac{P_{e}}{n(1+x)(x_{H_{2}}q_{\mathrm{LHV}}(H_{2})+x_{\mathrm{CO}}q_{\mathrm{LHV}}(\mathrm{CO})+x_{{\mathrm{CH}}_{4}}q_{\mathrm{LHV}}({\mathrm{CH}}_{4}))}\\ & =\frac{\left(x_{H_{2}}+4x_{{\mathrm{CH}}_{4}}+x_{\mathrm{CO}})u_{H_{2}}[-\Delta g(T)-n_{e}F(V_{act}+V_{ohm}+V_{con})\right]}{\left(1+x)(x_{H_{2}}q_{\mathrm{LHV}}(H_{2})+x_{\mathrm{CO}}q_{\mathrm{LHV}}(\mathrm{CO})+x_{{\mathrm{CH}}_{4}}q_{\mathrm{LHV}}({\mathrm{CH}}_{4})\right)}, \end{array}$$

where $q_{\mathrm{LHV}}(k)$ is the lower heating value of per molar k. Combining Equations (18)–(31), η is dependent on $n^{*}$, $u_{H_{2}}$, *x*, and T, while there is a linear relationship between $u_{H_{2}}$ and *x*. If temperature T is given, one can obtain the maximum efficiency varying with the molar flowing rate of the syngas by optimizing the hydrogen utilization factor in the fuel cell, as shown by the dash curve in Figure 7. The electric power density, hydrogen utilization factor, and x under the maximum efficiency are, respectively, shown by the dash curves in Figure 4, Figure 5 and Figure 6. If the values of $u_{H_{2}}$ and *x* derived in Section 3 are substituted into Equation (31), one can obtain the efficiency under the maximum power density as shown by the solid curve in Figure 7. The efficiency of the system is decreasing with the increase of the molar flowing rate of syngas.

When $n^{*}$ is smaller than 0.065 (mol·s^−1^·m^−2^), the working conditions of the maximum efficiency and the maximum power density are the same: The hydrogen should be reacted totally in the fuel cell, and the molar ratio of the syngas into the burner and steam reformer is 0.4. When $0.065\leq n^{*}\leq 0.14$ (mol·s^−1^·m^−2^), there should be residual hydrogen leaving the fuel cell and x is smaller than 0.4 for the maximum efficiency of the system. When $n^{*}$ is larger than 0.14 (mol·s^−1^·m^−2^), the hydrogen utilization factor is constant and about 0.61; there is no need to add syngas into the burner.

## 5. Conclusions

A model of the PEM fuel cell based syngas is established, and the residual hydrogen leaving the fuel cell and extra syngas are burned in a burner to supply enough high-temperature heat for the steam reforming reaction. There is a linear relationship between the hydrogen utilization factor in the fuel cell and the molar ratio of the syngas into the burner and steam reformer; if the hydrogen utilization factor is higher, the molar ratio is larger, and vice versa. Based on the thermodynamics and electrochemistry, the expressions of the electric power and energy conversion efficiency of the system are derived. For the maximum power density or maximum efficiency of a PEM fuel cell based syngas, the optimal hydrogen utilization factor in the fuel cell and the molar ratio of the syngas into the burner and steam reformer are, respectively, determined. In the future, the waste heat from a PEM fuel cell and the water gas shift reactions can be considered to be utilized to improve the energy conversion efficiency of the PEM fuel cell.

## Figures and Tables

**Table 1:** The composition of syngas [20].

Component k	$H_{2}$	${\mathrm{CH}}_{4}$	CO	${\mathrm{CO}}_{2}$	$H_{2}O$	$N_{2}$
Mole fraction of k in syngas: *x*_k_	0.13	0.01	0.16	0.05	0.36	0.29

**Table 2:** Thermodynamic parameters of the chemical components [21,22], where (g) and (l) refer to gas and liquid phases, respectively.

Component k	$h_{k}^{0}$ (J·mol^−1^)	$s_{k}^{0}$ (J·mol^−1^·K^−1^)	$L_{H_{2}O,m}$ (J·mol^−1^)	Molar Heat Capacity $C_{k,m}$ (J·mol^−1^·K^−1^)
N_2_	0	—	—	29.12
O_2_	0	205.138	—	25.8911 + 0.0129874t − 0.0000038644t^2^
CH_4_	−75,000	—	—	14.1555 + 0.0755466t − 0.0000180032t^2^
CO_2_	−393,800	—	—	26.0167 + 0.0435259t − 0.0000148422t^2^
CO	−110,500	—	—	26.8742 + 0.006971t − 0.0000008206t^2^
H_2_	0	130.695	—	29.0856 − 0.0008373t + 0.0000020138t^2^
H_2_O (g)	−241,800	—	—	30 + 0.01071t + 33000/t^2^
H_2_O (l)	−285,800	69.940	40,700	75.44

**Table 3:** Parameters used in the system.

Parameter	Value
Number of electrons, $n_{e}$	2
Faraday constant, F (C mol^−1^)	96485
Universal gas constant, R (J·mol·K^−1^)	8.314
Pressure at the anode, $p_{a}$ (atm)	3 [13]
Pressure at the cathode, $p_{c}$ (atm)	5 [13]
Anode stoichiometry, $\delta_{a}$	1.5 [20]
Cathode stoichiometry, $\delta_{c}$	3 [20]
Dry gas molar ratio at anode, $x_{a}$	$\left(x_{N_{2}}+x_{{\mathrm{CO}}_{2}}+x_{{\mathrm{CH}}_{4}}+x_{\mathrm{CO}})/(x_{H_{2}}+x_{\mathrm{CO}}+4x_{{\mathrm{CH}}_{4}}\right)$ [13]
Dry gas molar ratio at cathode, $x_{c}$	3.762 (air)
Charge transfer coefficient at the anode, $\alpha_{a}$	0.5 [20]
Charge transfer coefficient at the cathode, $\alpha_{c}$	1 [20]
Membrane thickness, $\delta_{mem}$ (cm)	0.018 [13]
$\mu_{mem}$	14 [21]
Constant, $\beta_{2}$	2 [20]
Limiting current density, $i_{L}$ (A cm^−2^)	2 [20]
T = 70 °C: $p_{s}$ (atm); $\beta_{1}$	0.3071; 0.2048
$q_{\mathrm{LHV}}(k)$ (kJ mol^−1^): k = H_2_; CO; CH_4_	241.9; 283.2; 803.7

## Nomenclature

$A_{c}$	Effective surface area of the bipolar plate
$\alpha_{a}$	Charge transfer coefficient at anode
$\alpha_{c}$	Charge transfer coefficient at cathode
$\beta_{1}$	Parameter in the expression of overpotential
$\beta_{2}$	Constant in the expression of overpotential
$C_{k,m}$	Molar heat capacity of k
Δh	Enthalpy change of gases
Δh(T)	Enthalpy change of gases
Δg(T)	Gibbs function change of gases
Δs(T)	Entropy change of gases
$\delta_{a}$	Stoichiometry coefficient
$\delta_{c}$	Stoichiometry coefficient
$\delta_{mem}$	Membrane thickness
F	Faraday constant
η	Efficiency of the hybrid system
$h_{k}$	Molar enthalpy of k at 600 °C
$h_{k}^{0}$	Molar enthalpy of k at 25 °C
$h_{k}(T)$	Molar enthalpy of k at temperature T
${\dot{H}}_{cell}$	Enthalpy of gases leaving the fuel cell
${\dot{H}}_{in}$	Enthalpy of gases into burner
${\dot{H}}_{out}$	Enthalpy of gases leaving the burner
I	Electric current of a PEM fuel cell
$i_{0}$	Exchange current density of the electrodes
i	Current density of a PEM fuel cell
$i_{L}$	Limiting current density of a PEM fuel cell
$L_{H_{2}O,m}$	Latent heat of one mole water
$\mu_{mem}$	Water content
n	Mole flow rate of syngas into HE1
$n_{H_{2}O}$	Mole rate of water added into syngas
$n^{*}$	Mole flow rate of syngas into HE1 unit area
$n_{e}$	Number of electrons
$P_{e}^{*}$	Electric power density of a PEM fuel cell
$P_{e}$	Electric power of a PEM fuel cell
$p_{c}$	Pressure at the cathode
$p_{a}$	Pressure at the anode
$p_{s}$	Saturation pressure of water
$p_{k}$	Partial pressure of k at the electrode
${\dot{Q}}_{2}$	The heat needed in HE2 unit time
${\dot{Q}}_{1}$	The heat needed in HE1 unit time
$q_{LHV}(k)$	Lower heating value of k per molar
R	Universal gas constant
$s_{k}^{0}$	Molar entropy of k at 25°C
$\sigma_{mem}$	Membrane conductivity
T	Operating temperature of a PEM fuel cell
$T_{C}$	Combustion temperature
t	Temperature
$T_{C}^{\prime }$	Temperature of gases leaving HE1
$u_{H_{2}}$	Hydrogen utilization factor in a fuel cell
$V_{act}$	Activation overpotential
$V_{ohm}$	Ohm overpotential
$V_{con}$	Concentration overpotential
$x_{c}$	Dry gas molar ratio at cathode
$x_{a}$	Dry gas molar ratio at anode
x	The molar ratio
$x_{k}$	Molar fraction of k in syngas
